# Etoricoxib in the treatment of osteoarthritis over 52-weeks: a double-blind, active-comparator controlled trial [NCT00242489]

**DOI:** 10.1186/1471-2474-6-58

**Published:** 2005-12-01

**Authors:** Sean P Curtis, Barry Bockow, Chester Fisher, Joseph Olaleye, Amy Compton, Amy T Ko, Alise S Reicin

**Affiliations:** 1 Merck & Co., Inc, Rahway, NJ, USA; 2 Arthritis Northwest, Seattle, WA, USA; 3 Clinical Research, Health Research of Hampton Roads, Newport News, VA, USA

## Abstract

**Background:**

The aim of this study was to evaluate the long-term efficacy and tolerability of etoricoxib, a COX-2 selective inhibitor, in osteoarthritis (OA) patients.

**Methods:**

A double-blind, randomized, multicenter study was conducted in 617 patients with OA of the knee. The base study was 14 weeks in duration and consisted of 2 parts; in Part I (6 weeks), patients were allocated to once daily oral etoricoxib 5, 10, 30, 60, 90 mg or placebo. In Part II (8 weeks); the placebo, etoricoxib 5 and 10 mg groups were reallocated to etoricoxib 30, 60, or 90 mg qd or diclofenac 50 mg t.i.d. Treatment was continued for consecutive 12 and 26 week extensions. Primary efficacy endpoints were the WOMAC VA 3.0 pain subscale and investigator global assessment of disease status. Safety and tolerability were assessed by collecting adverse events throughout the study.

**Results:**

Compared with placebo, the etoricoxib groups displayed significant (p < 0.05), dose-dependent efficacy for all primary endpoints in Part I; efficacy was maintained throughout the 52 weeks of the study. During the 46-week active-comparator controlled period, the etoricoxib groups demonstrated clinical efficacy that was similar to that of diclofenac 150 mg and was generally well tolerated, with a lower incidence of gastrointestinal (GI) nuisance symptoms compared with diclofenac (13.1, 14.7, and 13.5% for etoricoxib 30, 60, and 90 mg, respectively compared with 22.5% for diclofenac).

**Conclusion:**

In this extension study, etoricoxib, at doses ranging from 30 to 90 mg, demonstrated a maintenance of significant clinical efficacy in patients with OA through 52 weeks of treatment. Etoricoxib displayed clinical efficacy similar to diclofenac 150 mg and was generally well tolerated.

## Background

Osteoarthritis (OA) is a disorder that involves softening and disintegration of articular cartilage, vascular congestion and increased osteoblastic activity in the subarticular bone, new bone or cartilage growth at the joint margins, and capsular fibrosis. These factors often lead to pain, a common complaint among patients with OA. Other complaints include stiffness, swelling, deformation, and loss of function in affected joints. Appropriate treatment of OA is important in maintaining patients' mobility and overall quality of life as the disorder can lead to severe functional impairment and disability [1,2].

Patients with OA are commonly treated with analgesic agents that inhibit the cyclooxygenase (COX) enzymes. These treatments include nonselective nonsteroidal anti-inflammatory drugs (NSAIDs) and COX-2 selective inhibitors [3]. Two COX isoforms, COX-1 and COX-2, are recognized; NSAIDs and COX-2 selective inhibitors exert their analgesic action by inhibiting COX-2 while inhibition of COX-1 can lead to gastrointestinal (GI) toxicity. Etoricoxib is a COX-2 selective inhibitor with anti-inflammatory and analgesic efficacy comparable to nonselective NSAIDs in a number of disease and treatment settings [4].

In a previously reported, 2-part, 14-week placebo and active comparator controlled trial, etoricoxib demonstrated clinical improvement that was significantly superior to placebo and similar to diclofenac 50 mg tid [5]. Two consecutive study extensions were conducted following the base study for a total of 52 weeks of treatment. The purpose of this report is to evaluate the maintenance of efficacy and tolerability of etoricoxib over 52 weeks of treatment.

## Methods

This study (Protocol 007) was conducted in 55 centers in the United States. The study protocol and procedures were approved by local Institutional Review Boards of each study center. All patients gave written informed consent prior to participation in the original study and before continuation in each study extension.

### Entry criteria

Eligible patients for the base study were a minimum of 40 years old and had both clinical and radiographic evidence of OA of the knee with symptoms for at least 6 months prior to study entry, and met American Rheumatism Association (ARA) functional class I, II or III. All patients required NSAID therapy for 25 of the 30 days prior to the screening and were required to meet predefined clinical flare criteria in order to be eligible for allocation [5]. Patients were eligible for the extension studies if they successfully completed the original base study, and they had to have completed the first extension without protocol violation to be eligible for the second extension.

Exclusion criteria for the base study included: significant renal impairment (calculated creatinine clearance <30 mL/min); clinically significant abnormalities on screening physical or laboratory examinations; class III/IV angina or uncontrolled congestive heart failure; uncontrolled hypertension; stroke or a transient ischemic attack within 2 years; active hepatic disease; a history of recent neoplastic disease, acute meniscal injury to the study joint within 2 years of study entry; arthroscopy in the study joint within 6 months of study entry; weight in excess of 280 pounds (120 kg) or allergy to acetaminophen or conventional NSAIDs. Patients were also excluded if they required systemic corticosteroids, warfarin, low-dose aspirin or ticlopidine, or if they had required intra-articular steroids for joints other than the study joint within the month prior to study entry or to the study joint in the 2 months prior to study entry. For the extension periods reported here, patients who experienced an AE that led to discontinuation from study therapy during a previous treatment period or were considered protocol violators were excluded from participation.

### Study design

The base study was a 2-part, 14-week, parallel-group study which was followed by 2 consecutive, double-blind, active-comparator-controlled extensions (12 and 26 weeks, respectively). Part I of the base study was placebo-controlled; part II and the 2 extensions were active-comparator controlled. The base study was conducted to define the clinically active dose range of etoricoxib in patients with OA; the extension studies were conducted to evaluate the safety, tolerability and observe the efficacy of etoricoxib over an additional 38 weeks. With the base and extension studies, patients could have participated for a total of up to 52 weeks.

**Figure 1:**
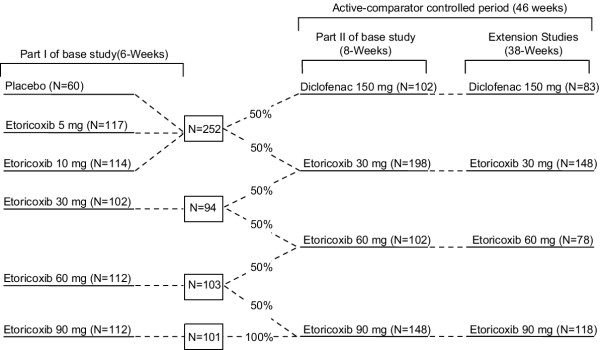
Study Design. The patient flow during the entire 52-week period is illustrated. The efficacy and safety evaluation for this report is based on data from the 46-week active-comparator controlled period.

Following discontinuation of previous therapy and a subsequent flare of clinical OA symptoms, eligible patients with OA of the knee were randomized to receive placebo, etoricoxib 5, 10, 30, 60, or 90 mg daily for 6 weeks (part I) according to a computer generated allocation schedule. Patients continued in Part II for an additional 8 weeks in which patients who were on placebo, etoricoxib 5, or 10 mg were given diclofenac 150 mg or etoricoxib 30 mg. Additionally, 50% of patients who were on etoricoxib 30 mg in part I continued on the same treatment while the other 50% were given etoricoxib 60 mg. Similarly, 50% of patients on etoricoxib 60 mg in part I continued on the same treatment while the other 50% were given etoricoxib 90 mg. Patients taking etoricoxib 90 mg in Part I continued on the same treatment (Figure 1). Upon completion of the 14 week base study, patients continued to receive the same treatment throughout the extension studies as in Part II (Figure 1). The treatment groups for Part I and II and all subsequent extensions were pre-assigned at randomization; blinding of patients and study staff was maintained throughout [5].

### Efficacy and safety assessments

The efficacy data reported here are for the 46-week active comparator controlled period (Weeks 6 to 52), during which efficacy assessments were made at Treatment Weeks 6, 8, 14, 20, 26, 34, 42, and 52. However, the efficacy over the entire 52-week period (base study and extension period) is also shown to assess the maintenance of effect over the entire treatment period for the subset of patients who received the same treatment for the entire 52-week period. Efficacy was evaluated using the following two primary endpoints for the active comparator controlled period: the Western Ontario and McMaster Universities OA index (WOMAC) VA 3.0 Pain Subscale (100 mm Visual Analog Scale [VAS] ;0 = no pain and 100 = extreme pain) and Investigator Global Assessment of Disease Status (0 – 4 point scale; where 0 = very well and 4 = very poor).

Safety and tolerability data reported here are also from the active-comparator controlled period for patients received the same study medication for up to 46 weeks, Week 6 to 52. As with efficacy, safety evaluations were made at study visits on Treatment Weeks 6, 8, 14, 20, 26, 34, 42, and 52. Vital signs were monitored at each of these visit. Laboratory studies including a complete blood count (CBC), serum chemistry panel, and urinalysis were performed at each visit. Clinical and laboratory AEs were recorded throughout the study; the investigator assessed the relation of AEs to study medication, the outcome of the AE, and any action taken. Identification and evaluation of AEs by the investigator, including those deemed to be "serious" according to regulatory definitions, were done while still blinded to study treatment.

### Statistical analysis

Treatment response was assessed mainly through interpretation of graphical presentations. For each efficacy endpoint, these graphs represented the least-squares mean (LS Mean) changes from baseline and the corresponding standard errors at each time point. LS mean changes were estimated from an analysis of covariance (ANCOVA) model with treatment sequence as the main factor and baseline value (at the Flare/Randomization Visit) as a covariate. The efficacy analyses were based on a modified intention-to-treat principle where all patients who entered the first extension, had a baseline value and at least one post-baseline measurement in the analysis period were included. For efficacy, missing values were imputed by the last value measured prior to that visit (last-value-carried-forward method). However, the efficacy values measured in part II of the base study were not carried forward to the first extension. The two extensions were assessed as one continuous treatment period, and all patients who entered the first extension were included in the efficacy and safety analyses.

The maintenance of efficacy over 52 weeks was evaluated based upon the subgroup of patients who received the same treatment and dose throughout the entire study (i.e. from the start of the base study to the end of the extension studies, which include only patients taking etoricoxib 30, 60, and 90 mg over 52 weeks). The LS mean changes from baseline (observed value at the Flare/Randomization visit of the base study) were plotted and tabulated for these 3 subgroups of patients from the Screening Visit to Week 52.

The following AEs were prespecified for evaluation: patients with one or more clinical AEs; drug-related clinical AEs; serious clinical AEs (i.e. any AE that results in death, is life threatening, results in persistent or significant disability, prolongs an existing inpatient hospitalization, or any event that jeopardizes the patient based on appropriate medical judgment); clinical AEs resulting in discontinuation of therapy; digestive system AEs (including abdominal pain) resulting in discontinuation of therapy; lower extremity edema AEs; discontinuations due to lower extremity edema AEs; hypertension AEs resulting in discontinuation of therapy; and AEs related to congestive heart failure, pulmonary edema, or cardiac failure. GI nuisance symptoms (i.e. abdominal pain, acid reflux, dyspepsia, epigastric discomfort, heartburn, nausea, and vomiting), adverse experiences that are common among patients who use nonselective NSAIDs, were examined. Also, laboratory parameters that were prespecified for evaluation included hemoglobin, hematocrit, alanine aminotransferase (ALT), aspartate aminotransferase (AST), and serum creatinine.

## Results

**Figure 2:**
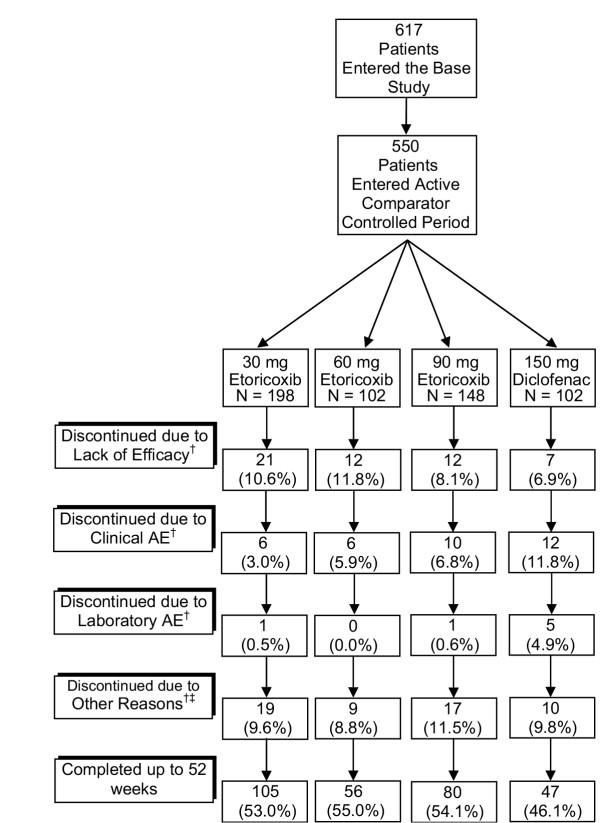
Patient accounting. ^† ^Number represents any patient who discontinued during the active comparator controlled period. Percent is calculated using the number of patients that entered the first extension as the denominator. ^‡ ^Other Reasons include lost to follow-up, patient moved, patient withdrew consent, or protocol deviation.

There were 617 patients enrolled into the base study. Of these 617 patients, 550 patients entered part II (the beginning of the active-comparator controlled period) and 427 patients completed Part II and entered the first extension. Among the 550 patients who entered the active comparator controlled period, 198, 102, 148, and 102 patients received 30, 60, and 90 mg etoricoxib daily and 50 mg diclofenac three times daily, respectively. At randomization, there were no clinically meaningful differences in baseline characteristics between the treatment groups (Table 1). The mean age was 62 years, 72% of the patients were women, and mean duration of OA of the knee was 7.4 years. During the active comparator controlled period, the most common reasons for discontinuation were clinical AEs (Figure 2).

**Table 1:** Baseline Patient Characteristics at Randomization (Patients Entering the Active Comparator Controlled Period – Weeks 6–52)

	Etoricoxib			Diclofenac
		
	30 mg	60 mg	90 mg	150 mg
	(N = 198)	(N = 102)	(N = 148)	(N = 102)
	n (%)	n (%)	n (%)	n(%)
**Gender**				
Female	141 (71.2)	75 (73.5)	100 (67.6)	79 (77.5)
Male	57 (28.8)	27 (26.5)	48 (32.4)	23 (22.5)
**Mean age in years (SD)**	61.9 (10.4)	62.3 (10.2)	60.6 (9.6)	62.3 (10.4)
**Race**				
White	176 (88.9)	89 (87.3)	131 (88.5)	93 (91.2)
Black	15 (7.6)	6 (5.9)	10 (6.8)	6 (5.9)
Hispanic	7 (3.5)	5 (4.9)	7 (4.7)	2 (2.0)
Native American	0 (0.0)	2 (2.0)	0 (0.0)	1 (1.0)
**Mean Duration of OA in years (SD)**	7.8 (7.9)	7.5 (6.6)	7.8 (7.4)	7.5 (7.1)
**ARA Function Class**				
Class I	25 (12.6)	12 (11.8)	25 (18.6)	19 (18.6)
Class II	134 (67.7)	72 (70.6)	95 (65.7)	67 (65.7)
Class III	39 (19.7)	18 (17.6)	27 (18.4)	16 (15.7)
**WOMAC Pain Subscale (100 mm VAS† [Mean (SD)]**	68.4 (17.0)	68.2 (17.7)	67.7 (16.9)	69.8 (16.1)
**Investigator Global Assessment of Disease Status (0 to 4 Likert Scale) [Mean (SD)]**	2.9 (0.6)	2.8 (0.7)	2.9 (0.6)	2.8 (0.7)

^† ^0 to 100 mm Visual analogue scale.

### Efficacy

**Figure 3:**
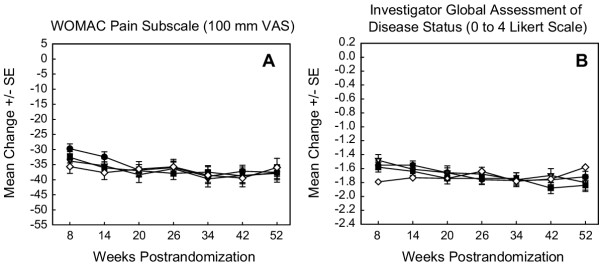
Primary efficacy endpoints from during the active comparator controlled period (weeks 6 to 52). This is a comparison of etoricoxib 30, 60, and 90 mg with diclofenac 150 mg during the active comparator controlled extension periods. LS Mean Change from Baseline (Randomization) is shown. Modified intention-to-treat approach with last value carried forward was used. The number of patients at later visits (≥34 weeks) was small. Data should, therefore, be interpreted with caution. SE = Standard error.  = 30 mg etoricoxib;  = 60 mg etoricoxib;  = 90 mg etoricoxib;  = 150 mg diclofenac.

Over the 46-week active comparator controlled period, etoricoxib 30, 60, and 90 mg had similar efficacy compared with diclofenac 150 mg (Figure 3). For both primary efficacy endpoints, the degree of clinical improvement relative to baseline was at or above the level of clinical importance, which was defined as 10 mm on the VAS WOMAC Pain Subscale or 0.5 Likert units on the Investigator Global Assessment of Disease Status [5]. Furthermore, this degree of improvement was maintained at a relatively constant level during the two extension periods for all active treatment groups (Figure 3).

**Figure 4:**
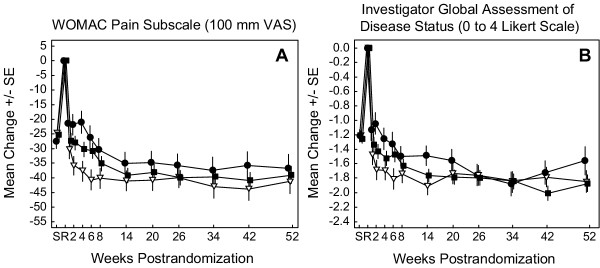
Primary endpoints evaluating efficacy of etoricoxib. LS Mean Change from Baseline is shown for patients receiving 30, 60 or 90 mg etoricoxib for up to 52 weeks. Modified Intention-to-Treat Approach With Last Value Carried Forward was performed. As the numbers of patients were small and decreased over time, data should be interpreted with caution. Screening (S) to baseline (R) = NSAID washout period; SE = Standard error ○ = 30 mg etoricoxib; * = 60 mg etoricoxib; ◆ = 90 mg etoricoxib.

To demonstrate the consistency of efficacy in patients on the same etoricoxib treatment over the 52-week treatment period, results for the subset of patients who received the same treatment for the entire 52-week period are also provided (Figure 4). The maximal treatment effect was reached during the base study at the end of Week 6 for the 30, 60, and 90 mg etoricoxib groups and maintained throughout the 52-week treatment period for the two primary endpoints, with small differences in efficacy favoring the 60 mg group [5].

### Safety

During the 46-week active-comparator controlled period, etoricoxib 30, 60, and 90 mg and diclofenac 150 mg were generally well tolerated, consistent with previously reported results for Part 1 of the base study (Table 2). The incidence of clinical AEs was similar among the treatment groups. Clinical AEs determined by the investigator (blinded) to be possibly, probably, or definitely drug-related were slightly greater in the etoricoxib 90 mg and diclofenac 150 mg groups (23.0% and 24.5%, respectively) compared with etoricoxib 30 mg (17.2%) and etoricoxib 60 mg (17.6%). More patients in the diclofenac group discontinued due to an AE (11.8%) compared with the etoricoxib groups (3.0 to 6.8%).

**Table 2:** Clinical Adverse Experience Summary During the Active-Comparator Period (Weeks 6–52)

	Etoricoxib 30 mg	Etoricoxib 60 mg	Etoricoxib 90 mg	Diclofenac 150 mg
	(N = 198)	(N = 102)	(N = 148)	(N = 102)
	n (%)	n (%)	n (%)	n (%)
**Clinical Adverse Experiences (AEs)**				
All Clinical AEs	136 (68.7)	73 (71.6)	112 (75.7)	81 (79.4)
Drug-related Clinical AEs^†^	34 (17.2)	18 (17.6)	34 (23.0)	25 (24.5)
Serious AEs	12 (6.1)	3 (2.9)	5 (3.4)	7 (6.9)
Discontinued due to an AE	6 (3.0)	6 (5.9)	10 (6.8)	12 (11.8)
				
Most Common Clinical AEs *^‡^				
Dizziness	6 (3.0)	6 (5.9)	6 (4.1)	1 (1.0)
Influenza-Like Disease	14 (7.1)	6 (5.9)	4 (2.7)	3 (2.9)
Upper Respiratory Infection	32 (16.2)	14 (13.7)	28 (18.9)	21 (20.6)
Diarrhea	12 (6.1)	1 (1.0)	7 (4.7)	13 (12.7)
Heartburn	6 (3.0)	5 (4.9)	7 (4.7)	6 (5.9)
Nausea	8 (4.0)	5 (4.9)	4 (2.7)	6 (5.9)
Headache	10 (5.1)	6 (5.9)	1 (0.7)	3 (2.9)
Urinary Tract Infection	10 (5.1)	3 (2.9)	5 (3.4)	5 (4.9)
				
**Gastrointestinal (GI) Nuisance Symptoms**^%^				
GI Nuisance Symptom AEs^%^	26 (13.1)	15 (14.7)	20 (13.5)	23 (22.5)
Discontinuations	0 (0.0)	1 (1.0)	2 (1.4)	4 (4.0)
				
**Renovascular AEs**				
Lower Extremity Edema	9 (4.5)	4 (3.9)	5 (3.4)	2 (2.0)
Discontinuations	1 (0.7)	0 (0.0)	0 (0.0)	0 (0.0)
				
Congestive Heart failure	0 (0.0)	1 (1.3)	0 (0.0)	0 (0.0)
Discontinuations	0 (0.0)	0 (0.0)	0 (0.0)	0 (0.0)
				
Hypertension	4 (2.0)	3 (2.9)	8 (5.4)	5 (4.9)
Discontinuations	1 (0.7)	0 (0.0)	1 (0.8)	1 (1.2)

^†^Determined by the investigator to be possibly, probably, or definitely related to treatment

* Incidence 5.0% or more in any treatment group

^‡ ^Hypertension is among the most common AEs, but is listed under the Renovascular AEs section of the table.

^%^Includes abdominal pain, acid reflux, dyspepsia, epigastric discomfort, heartburn, nausea, and vomiting

A higher percentage of patients in the diclofenac group had GI nuisance symptoms (26.6%) than in the etoricoxib groups (16.5%, 16.7%, and 14.9% in the 30, 60, and 90 mg etoricoxib groups, respectively). Consistent with this, the percent of patients who discontinued due to these GI nuisance symptoms was higher in diclofenac (4.0%) than in the etoricoxib groups (0.0%, 1.0%, and 2.1% in the 30, 60, and 90 mg etoricoxib groups, respectively; Table 2). One patient (etoricoxib 90 mg) had a gastric ulcer during the 52 week period. This patient had a prior history of esophagitis and gastric erosion. There were no other reports of patients with GI perforations, ulcers, or bleeding events.

Lower extremity edema was reported for 4.5, 3.9, and 3.4% in the 30-, 60-, and 90-mg etoricoxib groups, respectively and 2.0% in the diclofenac group. There was evidence for a dose-related trend in the proportion of patients with hypertension: 2.0, 2.9, and 5.4% in the 30, 60, and 90 mg etoricoxib groups, respectively and 4.9% in the diclofenac group. One patient in the etoricoxib 30 mg group discontinued due to lower extremity edema and one patient in each of the following treatment groups discontinued due to hypertension: etoricoxib 30 and 90 mg and diclofenac. One patient (etoricoxib 60 mg) who had an AE that consisted of mild congestive heart failure and was felt by the investigator to not be drug related, continued on the study and recovered.

There was one confirmed CV thrombotic event during the active-comparator controlled period; a cerebrovascular accident occurred in a patient taking diclofenac 150 mg. The event was deemed to not be drug-related and the patient was discontinued from the study after being put on low-dose aspirin therapy (an excluded medication).

Laboratory AEs were reported for 5.6, 5.0, 9.6, and 18.6% in the 30, 60, and 90 mg etoricoxib and 150 mg diclofenac groups, respectively during the active-comparator controlled period (Table 3). Drug-related laboratory AEs were reported for 2.0, 0.0, 4.1, and 11.8 % in the 30, 60, and 90 mg etoricoxib and 150 mg diclofenac groups, respectively. The most frequently reported laboratory AEs were increased levels of ALT and AST, which accounted for much of the higher incidence of laboratory AEs in the diclofenac group. Elevated levels of these enzymes were reported in a greater percent of patients in the diclofenac group (10.8%) versus the etoricoxib groups (1.0 to 2.1%) (Table 3). Three patients on diclofenac discontinued due to ALT/AST increases compared with no patients in the etoricoxib group. The largest change from baseline in ALT was observed at Week 20 in the diclofenac group (42.4%). Mean ALT decreased in subsequent weeks in the diclofenac group (6.6% above baseline at Week 52). Mean AST was also higher in the diclofenac group vs. the etoricoxib groups, but decreased over time. Two patients on etoricoxib (1 in the 30 mg group and 1 in the 90 mg group) and one patient on diclofenac 150 mg discontinued due to an increase in serum creatinine. Serum creatinine returned to baseline following discontinuation of study medication in these three patients.

**Table 3:** Laboratory Adverse Experience Summary During the Active-Comparator Period (Weeks 6–52)

	Etoricoxib 30 mg	Etoricoxib 60 mg	Etoricoxib 90 mg	Diclofenac 150 mg
	(N = 197)	(N = 101)	(N = 146)	(N = 102)
	n (%)	n (%)	n (%)	n (%)
**Laboratory AEs**				
Patients with one or more laboratory AEs	11 (5.6)	5 (5.0)	14 (9.6)	19 (18.6)
With drug-related adverse experiences^†^	4 (2.0)	0 (0.0)	6 (4.1)	12 (11.8)
With serious adverse experiences	0 (0.0)	0 (0.0)	0 (0.0)	0 (0.0)
Discontinued due to laboratory AEs	1 (0.5)	0 (0.0)	1 (0.7)	5 (4.9)
**Laboratory AEs of special interest**				
Alanine Aminotransferase Increased (ALT)	3 (1.5)	1 (1.0)	3 (2.1)	11 (10.8)
Aspartate Aminotransferase Increased (AST)	2 (1.0)	1 (1.0)	2 (1.4)	11 (10.8)
Hemoglobin Decreased	1 (0.5)	0 (0.0)	1 (0.7)	3 (2.9)
Hematocrit Decreased	1 (0.5)	0 (0.0)	1 (0.7)	1 (1.0)
Serum Creatinine Increased	1 (0.5)	1 (1.0)	2 (1.4)	2 (2.0)

^†^Determined by the investigator to be possibly, probably, or definitely related to treatment

In addition to the analysis of patients during the active-comparator controlled period, safety data is presented showing rates of most common AEs and AEs of special interest (GI and renovascular AEs) per 100 patient-years for all patients treated with etoricoxib 30, 60, or 90 mg or diclofenac in either the placebo-controlled or active comparator-controlled periods (this includes patients that received a different treatment during the placebo-controlled period) (Table 4). The AEs occurring at the highest rate were upper respiratory infection, headache, and diarrhea. Lower extremity edema occurred at a higher rate in the etoricoxib groups compared with diclofenac. Hypertension occurred at a higher rate in the etoricoxib 90 mg and diclofenac groups compared with the etoricoxib 30 mg and 60 mg groups.

**Table 4:** Rates of Adverse Experiences (AEs) of Special Interest per 100 Patient-YearsAll Patients Over The Entire 52 Weeks From The Start of Therapy to the End of the Period

		Etoricoxib		Diclofenac
	30 mg	60 mg	90 mg	150 mg
	n (rate^†^)	n (rate^†^)	n (rate^†^)	n (rate^†^)
Most Common Clinical AEs*				
Diarrhea	20 (15.4)	9 (12.2)	14 (13.4)	14 (23.2)
Dizziness	9 (6.9)	8 (10.8)	9 (8.6)	1 (1.7)
Dyspepsia	8 (6.1)	3 (4.1)	5 (4.8)	8 (13.3)
Headache	19 (14.6)	18 (24.4)	4 (3.8)	4 (6.6)
Influenza-like disease	18 (13.8)	7 (9.5)	6 (5.8)	4 (6.6)
Lower Extremity Edema	11 (8.4)	9 (12.2)	7 (6.7)	2 (3.3)
Nausea	14 (10.7)	9 (12.2)	10 (9.6)	7 (11.6)
Rash	6 (4.6)	10 (13.5)	4 (3.8)	1 (1.7)
Sinusitis	15 (11.5)	5 (6.8)	14 (13.4)	5 (8.3)
Upper Respiratory Infection	45 (34.5)	31 (42.0)	41 (39.3)	26 (43.1)
				
**GI Nuissance Symptoms**				
Abdominal pain	11 (8.4)	4 (5.4)	2 (1.9)	5 (8.3)
Acid reflux	0 (0)	0 (0)	0 (0)	1 (1.7)
Dyspepsia	8 (6.1)	3 (4.1)	5 (4.8)	8 (13.3)
Epigastric discomfort	4 (3.1)	1 (1.4)	6 (5.8)	2 (3.3)
Heartburn	7 (5.4)	7 (9.5)	9 (8.6)	6 (9.9)
Nausea	14 (10.7)	9 (12.2)	10 (9.6)	7 (11.6)
Vomiting	3 (2.3)	1 (1.4)	0 (0)	1 (1.7)
				
**Renovascular AEs**				
Lower extremity edema	11 (8.4)	9 (12.2)	7 (6.7)	2 (3.3)
Congestive heart failure	0 (0)	2 (2.7)	0 (0)	0 (0)
Hypertension	7 (5.4)	4 (5.4)	10 (9.6)	5 (8.3)

*Rate ≥ 10% in any group

^† ^Rate per 100 patient years of exposure = (# of AEs / Patient Years)

*100 n = # of AEs

## Discussion

Etoricoxib 30, 60 and 90 mg demonstrated clinical efficacy in patients with OA in the first 6 weeks of therapy [5]; this level of efficacy was maintained during the 46-week active comparator controlled period. The magnitude of improvement observed with the etoricoxib 60 and 90 mg groups was similar to that of the diclofenac group; the efficacy demonstrated by the etoricoxib 60 and 90 mg groups was slightly greater than that observed with the etoricoxib 30 mg group. This was most evident in the efficacy evaluation of patients treated with the same dose of etoricoxib from the beginning of the study through the second extension. All treatments were generally well tolerated and no new or unique findings related to safety or tolerability were revealed during long-term dosing of etoricoxib (i.e., over 52 weeks).

Patients who received the same dose of etoricoxib from the beginning of the base study through the second extension demonstrated that, generally, the treatment effect reached a plateau at approximately week 6 of therapy; the treatment effect was maintained at that level through week 52 of the study.

In the placebo-controlled period (part I) of the base study, the treatment responses of etoricoxib 30 mg were significantly greater than that of placebo, but were approximately one-half to two-thirds of that of etoricoxib 60 mg as assessed by the primary endpoints. Etoricoxib 60 and 90 mg provided similar efficacy, indicating that the 60 mg dose was the minimal dose with maximal efficacy [5]. During the extension studies, however, patients who continued on the 30 mg dose had a treatment response that more closely approximated that of the 60 mg and 90 mg doses; the treatment effect for all treatment groups was maintained for up to 52 weeks. Furthermore, the treatment effect with etoricoxib (30, 60 and 90 mg) was similar to that of diclofenac 150 mg. These results should be interpreted cautiously as the number of patients in each of the treatment groups was relatively small and decreased over time due to discontinuations from the study.

Both etoricoxib and diclofenac appeared to be well-tolerated. However, patients in the etoricoxib group experienced fewer GI nuisance symptoms during the extensions. A larger proportion of patients in the diclofenac group discontinued due to these GI nuisance symptoms. In 8 previous clinical trials, less patients on etoricoxib discontinued due to a GI symptom vs. comparator nonselective NSAIDs [6]. In one randomized controlled trial, patients receiving etoricoxib had less fecal blood loss versus ibuprofen and in another trial, etoricoxib patients had fewer endoscopically detectable lesions versus naproxen; these data suggest that etoricoxib may be associated with a reduced incidence of GI perforations, ulcers, or bleeds (PUBs) [7]. Additionally, in a large clinical trial, etoricoxib 90 mg demonstrated a superior GI tolerability profile compared with diclofenac 150 mg; there were significantly fewer patients on etoricoxib that experienced a GI AE versus diclofenac [8].

There was only one serious thrombotic cardiovascular (CV) event during the active comparator-controlled period, which occurred in a patient receiving diclofenac. In this study, use of low dose aspirin, which is a factor that is indicative of CV risk, was not allowed since the study was initiated before the GI COX-2 hypothesis was proven. Although aspirin users were excluded from the study, patients with other cardiovascular risk factors (i.e., chest pain, hypertension, history of smoking, hypercholesterolemia, palpitation, mitral valve prolapse, and sinus bradycardia) were allowed in the study.

To assess either GI or CV safety, larger and longer-term trials or pooled analyses are required since the incidence of such events is relatively rare [9,10]. In a pooled analysis of the etoricoxib development program, the rate of serious thrombotic CV events in patients treated with etoricoxib was not discernibly different than in patients treated with NSAIDs that are not associated with potent and sustained antiplatelet effects such as diclofenac and ibuprofen [11]. Although no discernible difference was observed in comparison to placebo either, the placebo-controlled thrombotic CV safety data for etoricoxib are limited in quantity and in duration to 12 weeks. However, results from longer-term, placebo-controlled trials with rofeocoxib and celecoxib suggest that use of COX-2 selective inhibitors are associated with an increased incidence of adverse CV events compared to placeb [12,13]. Large, long-term trials to assess the CV safety profile of etoricoxib are ongoing.

Renovascular effects were also examined during the 52-week treatment period since NSAIDs and COX-2 selective inhibitors can affect renal physiology [14,15]. In the base study, there was a small number of renovascular AEs; most common with the 90-mg dose, minimal with the 60-mg dose, and not detectable with 30 mg etoricoxib [5]. Overall, renovascular effects were of limited clinical significance during the active comparator periods. The incidence of congestive heart failure was low (1 event in a patient receiving etoricoxib 60 mg) and the incidence of hypertension with etoricoxib was generally similar to that of diclofenac, although the number of hypertension events was dose-related. The risks for renovascular effects in prior trials of the etoricoxib clinical development program were low with a shallow dose response and were generally similar to those found with naproxen or ibuprofen [16].

## Conclusion

In summary, etoricoxib once daily provided a clinical effect that was similar to that of diclofenac 150 mg in patients with OA and was maintained for up to 52 weeks. In the base study [5], etoricoxib 60 mg was the minimal dose with maximal efficacy; however, in this extension study, all thee doses of etoricoxib, including 30 mg, provided robust efficacy that was maintained for up to 52 weeks. All treatments were generally well tolerated over 52 weeks. These data support the role of etoricoxib as an important long-term treatment option for the management of patients with OA.

## List of abbreviations

Abbreviation Definition

OA Osteoarthritis

COX Cyclooxygenase

NSAID Nonsteroidal anti-inflammatory drug

WOMAC Western Ontario and McMaster Universities OA index VA 3.0 Pain Subscale

VAS Visual Analog Scale

GI Gastrointestinal

CV Cardiovascular

AE Adverse experience

ALT Alanine aminotransferase

AST Aspartate aminotransferase

ARA American Rheumatism Association

CBC Complete blood count

LS Least squares

ANCOVA Analysis of covariance

PUB Perforation, ulcer, or bleed

## Competing interests

This study was funded by Merck & Co., Inc. Some of the authors are employees of and potentially own stock options for Merck & Co., Inc.

## Authors' contributions

SPC was the clinical monitor for this study. BB and CF were primary investigators. JO assisted in the monitoring of the study. AC assisted in writing the study report for the trial and provided editorial assistance with the manuscript. ATK provided statistical support. ASR provided guidance during the monitoring of the study and writing of the manuscript. All authors provided input in the preparation of the manuscript.

## Pre-publication history

The pre-publication history for this paper can be accessed here:


